# Comparative analysis of two methods for measuring sales volumes during malaria medicine outlet surveys

**DOI:** 10.1186/1475-2875-12-311

**Published:** 2013-09-05

**Authors:** Edith Patouillard, Immo Kleinschmidt, Kara Hanson, Sochea Pok, Benjamin Palafox, Sarah Tougher, Kate O’Connell, Catherine Goodman

**Affiliations:** 1 London School of Hygiene and Tropical Medicine, Keppel Street, London WC1E 7HT, UK; 2 Population Services International Cambodia, No. 29 Street 334, P.O. Box 153, Chamcar Mon BKK1, Phnom Penh, Kingdom of Cambodia; 3 Population Services International (PSI), Malaria & Child Survival Department, P.O. Box, 14355–00800, Nairobi, Kenya

**Keywords:** Medicines, Retail and wholesale outlet survey, Sales volumes, Measurement methods, Comparative analysis

## Abstract

**Background:**

There is increased interest in using commercial providers for improving access to quality malaria treatment. Understanding their current role is an essential first step, notably in terms of the volume of diagnostics and anti-malarials they sell. Sales volume data can be used to measure the importance of different provider and product types, frequency of parasitological diagnosis and impact of interventions. Several methods for measuring sales volumes are available, yet all have methodological challenges and evidence is lacking on the comparability of different methods.

**Methods:**

Using sales volume data on anti-malarials and rapid diagnostic tests (RDTs) for malaria collected through provider recall (RC) and retail audits (RA), this study measures the degree of agreement between the two methods at wholesale and retail commercial providers in Cambodia following the Bland-Altman approach. Relative strengths and weaknesses of the methods were also investigated through qualitative research with fieldworkers.

**Results:**

A total of 67 wholesalers and 107 retailers were sampled. Wholesale sales volumes were estimated through both methods for 62 anti-malarials and 23 RDTs and retail volumes for 113 anti-malarials and 33 RDTs. At wholesale outlets, RA estimates for anti-malarial sales were on average higher than RC estimates (mean difference of four adult equivalent treatment doses (95% CI 0.6-7.2)), equivalent to 30% of mean sales volumes. For RDTs at wholesalers, the between-method mean difference was not statistically significant (one test, 95% CI −6.0-4.0). At retail outlets, between-method differences for both anti-malarials and RDTs increased with larger volumes being measured, so mean differences were not a meaningful measure of agreement between the methods. Qualitative research revealed that in Cambodia where sales volumes are small, RC had key advantages: providers were perceived to remember more easily their sales volumes and find RC less invasive; fieldworkers found it more convenient; and it was cheaper to implement than RA.

**Discussion/conclusions:**

Both RA and RC had implementation challenges and were prone to data collection errors. Choice of empirical methods is likely to have important implications for data quality depending on the study context.

## Background

In many low- and middle-income countries, the private commercial sector plays an important role in the provision of malaria treatment [1-6]. A study on the market for anti-malarial drugs in six developing countries found that private providers were responsible for around 40% of all anti-malarial sales volumes in Zambia, 55% in Uganda, 71% in Cambodia, and 75% in both Benin and the Democratic Republic of Congo and 98% in Nigeria [2]. Their popularity is commonly attributed to convenience as they tend to operate closer to homes [7-10], and availability and reliability of drug stocks compared to public health providers [8,9,11-14]. Private providers vary substantially within and across countries and can include hospitals, clinics, pharmacies, drug shops, grocery stores, village shops, market stalls and mobile providers.

Given the importance of private commercial outlets, there has been increased interest in analysing their role in treatment provision and how this can be improved. A key aspect of this is the measurement of anti-malarial and diagnostic sales volumes. Sales volumes data can be used to calculate market shares and provide information on the relative importance of public and private sectors and how this varies across countries. These data can also be used to estimate the share of recommended first-line drugs or banned drugs, such as oral artemisinin monotherapy, sold in the market [1,2,15]. Data on sales of rapid diagnostic test kits for malaria can indicate the low frequency of parasitological-based diagnosis of malaria fevers [1]. Anti-malarial sales volumes have been measured to evaluate the effect of major drug subsidy programmes, such as the Affordable Medicine Facility-malaria (AMFm) for which the change in the market share of quality assured artemisinin combination therapy (ACT) was one of four key success metrics, alongside availability, price and use [15]. Finally, market share data can be used for assessing the nature of competition in the market for malaria treatment and its relationship with key retail market outcomes, notably anti-malarial prices and price mark-ups. For example, research on the market for malaria treatment in Tanzania and Cambodia has shown that market concentration measured using the Hirshman-Herfindahl index (HHI) (the sum of squared market shares of each firm in the market) is related to the extent to which providers can influence the price of anti-malarial drugs sold in the market [16,17].

A number of methods for measuring sales volumes have been identified, namely reviewing providers’ sales records, asking providers to recall their sales volumes over a given period, conducting exit interviews with customers, and retail audits. Retail audits involve visiting a panel of outlets to collect stock information at regular intervals; at each visit, fieldworkers measure the stocks of an entire product category and ask the shopkeeper about any volumes added and/or disposed of during the visit interval. The volume of sales for each shop during the period is then estimated by subtracting the stock at the end of the period from the stock at the initial visit, corrected by any additions/disposals during the period.

These data collection methods each have methodological challenges for collecting commercial sales volume data at private businesses in developing country settings. Providers’ sales records may be non-existent, incomplete and/or outdated. Private commercial providers may be reluctant to share their sales records as they may fear that these could be disclosed to drug regulation bodies, revenue authorities or competitors. Asking providers to recall their volumes is a convenient and popular method used in many surveys [18], but it may be prone to recall bias. Providers may also be unwilling to record or recall the sales of products that they are not authorized to handle. The retail audit approach could be perceived to provide more accurate responses as it does not rely on respondents’ ability to remember their sales volumes. However, as it requires at least two visits to the same outlets within a given period it is likely to be more costly and logistically complex than relying on records or provider recall. Exit interviews may be another approach to address this problem, although the presence of interviewers may bias sales patterns. Shopkeepers may also not allow interviewers to stand outside their shops or consumers may be reluctant to share information about their purchases or they may be in a rush leaving the shop. Overall, the available approaches tend to be better suited for estimating the sales of licensed rather than unlicensed outlets [19] and sales of registered rather than unregistered products.

Whilst the challenges of these different methods have been identified, what is lacking is evidence on how sales volume estimates collected through different methods in the same context compare. Furthermore, the available literature concentrates mainly on retail medicine providers, yet drug retailers are the last link in a chain of suppliers, including several layers of wholesalers. Much less attention has been paid to this market segment [20] and guidance on how to study wholesalers, notably on how best to collect their sales volume data, is scarce [17,18].

Using sales volume data collected at retail and wholesale outlets through two different methods, namely provider recall and retail audits, this paper provides evidence on the degree of agreement between the two methods in Cambodia. The study was undertaken with retail and wholesale providers as part of the ACTwatch project [18], which measured sales volumes using the recall method. Retail audits were selected as the comparative method because they had been used for measuring retail anti-malarial sales volumes in previous studies [16,21]. Written records were not included in the study due to their rarity among less formal providers. Exit interviews were not included because anti-malarials represented a small share of wholesalers’ total business [17], implying that interviewers may have had to wait many days outside a shop before identifying a wholesale anti-malarial customer.

At the time of the study, there were three categories of licensed medicine outlets in Cambodia, including pharmacies that were managed by a pharmacist, and dépots managed by an assistant pharmacist (dépot A) or a retired public health staff member (dépot B) with a minimum qualification of nurse or midwife [17]. Pharmacies were authorized to engage in both wholesale and retail activities whilst dépots were authorized to retail only [17]. Licensed providers were authorized to sell registered pharmaceutical drugs, hygienic and cosmetic products with preventive and curative properties, and dental, laboratory and medical equipment. At the time of the study, there were no clear regulations on the sales of malaria diagnostics, such as rapid diagnostics tests (RDTs). Around 520 pharmacies and 695 dépots were estimated to operate in Cambodia, supplemented by many other medicine sellers that operated illegally, including unlicensed pharmacies and drug shops that sold medicines, cosmetics and household goods; private clinics (sometime referred to as cabinets or clinical pharmacies) that sold medicines and also provided outpatient and/or inpatient clinical services; mobile providers who travelled to patients’ homes to provide clinical services, and at times offered outpatient and/or inpatient care at fixed outlets; and, grocery and village shops that sold medicines alongside food, soft drinks and other consumer goods [1,22].

## Methods

Sales volume data for anti-malarials and RDTs were collected through the recall (RC) and the retail audit (RA) methods, together referred to as the sales level surveys (SLS) in retail and wholesale commercial anti-malarial providers. The aim of the SLS was to explore whether RC and RA for measuring sales volumes agreed sufficiently that they can be used interchangeably. The relative strengths and weaknesses of each method were also analysed from an implementation perspective using qualitative methods.

### The sales level surveys

RA consisted of visiting each sampled outlet two times with a two-week time interval between each visit. At the first visit, referred to as the sales level survey 1 (SLS1), data on quantities stocked of each product were collected. At the second visit (SLS2), data on quantities stocked, quantities delivered between first (SLS1) and second (SLS2) visits, and quantities thrown away/transferred to other shops or sent back to wholesalers or confiscated were collected for each product in stock, including products in stock at either or both visits. Quantities stocked were physically counted where possible or providers were asked to state the quantities in stock. To collect data on quantities delivered and disposed of, providers were asked to check any available written records or sales receipts and in the absence of records, to recall these quantities. RC consisted of asking retailers and wholesalers to recall the quantities sold during the two-week time interval between SLS1 and SLS2. It was implemented at the start of SLS2 before collecting stock data in order to minimize bias as recall data may have been influenced by the process of counting stocks for the RA. A time interval of two weeks between the two visits at each outlet was chosen based on the existing literature in which a two-week time interval was considered reasonable for capturing wholesale deliveries [16,21]. A recall period of two weeks has also generally been used for collecting data such as fever episodes in household surveys [23-25].

The SLS sampling strategy drew on data collected during the ACTwatch retail outlet and supply chain surveys, which are described in detail elsewhere [18]. Briefly, for the ACTwatch retail outlet survey, a sample of 38 administrative clusters (health centre areas with catchment populations of 10–15,000 inhabitants) were selected with probability proportional to size from all 255 malaria endemic clusters in Cambodia [1]. Then, a census of all public and private outlets in these 38 clusters was completed and a list of those stocking anti-malarial drugs was created [1]. At each retail outlet, data were collected on the two most important wholesale supply sources for anti-malarial drugs. All anti-malarial wholesale supply sources mentioned by retailers were visited during the ACTwatch supply chain study. Data were collected on the two most important wholesale supply sources. This process was then repeated until the top of the chain was reached.

For the SLS, retail and wholesale outlets were purposively sampled from the list of anti-malarial retailers and wholesalers surveyed during the ACTwatch surveys. The geographical location of each commercial outlet and the number of outlets stocking anti-malarials in each location at the time of the outlet survey were used to select areas in which all outlets could be visited two times with a two-week time interval (in order to conduct the RA component).

A total of 107 retailers and 67 wholesale outlets were sampled. Wholesale and retail outlets not found, not stocking anti-malarials or not available at the time of the SLS were not replaced.

At retail level, the SLS was conducted as a standalone survey few months after the ACTwatch retail outlet survey. At wholesale level, the SLS took place during the ACTwatch supply chain survey. The questions relating to SLS1 (questions about quantities stocked) were asked after the supply chain survey questionnaire was administered, whilst SLS2 (questions about recall sales volumes, quantities stocked and quantities received and disposed of) was conducted two weeks later as a standalone survey.

All data collection tools were translated from English to Khmer and piloted before the start of data collection. A team of two interviewers entered each business, informed shopkeepers about the study objective and obtained consent. Interviews were conducted in Khmer, with the person most involved in the management of the business. Interviews were conducted in the premises, with breaks each time a customer arrived. Interviewers then asked whether they could return after two weeks and if so they arranged an appointment, and returned on that date.

All types of anti-malarial drugs in all dosage forms and packaging types, and RDTs were surveyed. For anti-malarials, data were collected in terms of both full packs and loose tablets (ie, those kept in containers/tins). Stock data for anti-malarials stored in half-full containers were estimated based on the height of the tablets in the pot measured using a ruler and the number of tablets in a full pot. RDT data were collected in terms of single RDT units.

For each anti-malarial observation, volume estimates were converted into adult equivalent treatment doses (AETDs) [1]. One AETD was defined as the amount of the drug needed for a full adult course of treatment based on guidelines from the World Health Organization (WHO) where available, or else from peer-reviewed literature or manufacturers. Anti-malarials missing data required to calculate AETDs (eg, drug strength) were excluded from the sales volume estimation [1].

RA estimates were calculated as: (total quantities stocked at SLS1) + (quantities delivered between SLS1 and SLS2) – (quantities disposed of between SLS1 and SLS2) – (total quantities stocked at SLS2).

Negative RA estimates indicating data collection errors during the SLS and anti-malarial/RDT observations without both RA and RC estimates were excluded from the analysis.

In outlets with sales data for more than one type of anti-malarial/RDT the sum of all RC estimates and of all RA estimates was calculated in order to obtain for each outlet single total sales volume estimates with each method.

The level of agreement between the two methods was explored following the Bland-Altman approach [26,27].

The first step was to calculate, for each outlet, the difference between RA and RC sales volume estimates for outlet _i_. Formally:

(a) $$RA_{i}‒RC_{i}$$

where RA_i_ and RC_i_ are sales volumes estimated through the two different methods at outlet _i_.

The second step was to estimate the “bias of the measurement” between the two methods, which is the mean of the differences between the two different methods (b1), and its standard deviation (SD) (b2). Formally:

(b1) $$\bar{\mathrm{RA}‒\mathrm{RC}}=\frac{1}{n}\sum {}_{i}^{n}\left(RA_{i}‒RC_{i}\right)$$

(b2) $$\mathrm{SD}=\sqrt{\frac{1}{n‒1}\sum {\left(X_{i}‒\bar{X}\right)}^{2}}$$

where $x_{i}=\left(RA_{i}‒RC_{i}\right)$ is the difference between RA and RC in outlet_i_$\bar{x}=\bar{\mathrm{RA}‒\mathrm{RC}}$ the mean of the differences between RA and RC across all outlets and n the total number of outlets with a pair of RA and RC estimates.

Differences between sales volume estimates were plotted on a histogram (not shown) to verify that they were approximately normally distributed.

The third step was to investigate for each outlet whether there was an association between the total volume sold and the bias (ie, the mean of the difference, b1). This is because for the bias to be a meaningful estimate of the level of agreement between the two different methods, it should be constant throughout the range of measurements [26,27]. In the absence of a recognized gold standard method for measuring sales volumes, an outlet’s “true” total sales volume was proxied as the mean of RC and RA estimates. Formally:

(c) $$\frac{RA_{i}+RC_{i}}{2}$$

The association between total volume sold (c) and measurement bias (b1) was explored graphically using a scatter plot of the differences against total volume sold and confirmed statistically using a correlation coefficient obtained through the STATA command *baplot*[28].

The fourth step was to calculate the interval within which 95% of paired estimates were expected to lie, referred to as the upper and lower limits of agreement (LoA) between the two methods [26]. Formally:

(d) $$\mathrm{LoA}=\bar{\mathrm{RA}‒\mathrm{RC}}\pm 1.96\mathrm{SD}$$

### Qualitative methods

The quantitative analysis was supplemented by qualitative data on information about the implementation process of RC and RA. Qualitative data were drawn from fieldworkers ‘diaries, which had been completed at the end of each outlet visit. In each diary, fieldworkers described and compared their experiences in collecting data across RC and RA, products, dosage forms and packaging types. They also recorded observations of shopkeepers’ behaviour during data collection.

Semi-formal group discussions were also organized during the course of the fieldwork to clarify diary entries. These discussions provided a forum for fieldworkers to elaborate on particular topics, share arduous experiences, discuss their views and trade funny stories. Group discussions also had the advantage of creating interactions between fieldworkers, which prompted others to remember their own experiences [29]. Group discussions were facilitated in English and/or Khmer by the corresponding author with the assistance of a trained Cambodian research assistant and recorded using written notes. Five group discussions were conducted at the mid and end of data collection with each of the three fieldworker teams involved in the SLS. Fieldwork diaries kept in Khmer were translated into English by a trained research assistant. These data were analysed using a simple thematic content approach through which recurrent themes under each of the topics discussed were listed and compared.

### Ethics considerations

The study received ethics clearance from the Cambodian National Ethics Committee for Health Research (no. 041 NECHR) and ethics review committee of the LSHTM (no. 5466).

Informed consent from each interviewed shopkeeper was obtained at SLS1 to cover both SLS visits. For diaries and group discussions, fieldworkers’ participation as research subjects was explained during the recruitment process and consent received orally from each fieldworker recruited.

## Results

### Quantitative results: Bland-Altman approach

Of the 67 wholesalers and 107 retailers initially sampled, 58 and 62% participated in the SLS, respectively. Reasons for non-participation at both wholesale and retail outlets at SLS1 included outlets not found, not open at the time of visit or not stocking anti-malarial drugs, whilst at SLS2 the main reason for non-participation was provider refusal. The SLS wholesale sample was similar to that surveyed during the nationally representative ACTwatch supply chain survey: outlets had a median of two workers (IQR 2–2), had been in operation for 10 years (IQR 4–13) and around 70% employed a member of staff with health qualifications, with nurse/midwife being the most commonly reported qualification type [17]. The SLS sample of retailers included pharmacies/clinical pharmacies (13%), drug shops (21%), mobile providers (20%), grocery stores (26%) and village shops (20%). Retailers shared similar characteristics with those of the commercial outlets interviewed during the ACTwatch outlet survey: staff with health qualifications were more commonly found at pharmacies (85%), drug shops (76%) and mobile providers (70%) than at grocery and village shops (13 and 12%, respectively), and the most commonly reported health qualifications were nurses/midwives. A median of two people (IQR 1–2) worked at the sampled outlets and shops had been in operation for a median of eight years (IQR 2–15) [17]. Surveyed anti-malarials were found in tablet and injectable forms only. Tablets were commonly stocked in packs, and injectables in individual ampoules. Tablets kept in opened tins/containers were rare and found at retail outlets only.

At wholesale outlets, 104 different anti-malarial products were surveyed. Sales volumes were collected for 76 anti-malarial products through RC and for 82 through RA (Table 1). For RDTs, 34 different products were surveyed and sales volumes were collected for 26 products through the RC and for 29 through the RA. The main reasons for non-response included wholesalers’ refusal to recall their sales volumes for the RC, and for the RA wholesalers’ refusal to let interviewers record stock data (Table 1). Wholesale sales volumes were estimated through both RC and RA for 62 anti-malarials and 23 RDTs.

**Table 1:** Data collected on wholesale and retail sales volumes using recall and retail audit methods

	**Number of products surveyed (%)**^**1**^			
	**Wholesale SLS**		**Retail SLS**	
	**Anti-malarials**	**RDT**	**Anti-malarials**	**RDT**
Total products surveyed	104 (100%)	34 (100%)	143 (100%)	42 (100%)
Recall method (RC)				
Sales volume data collected	76 (73.1%)	26 (76.5%)	130 (91.0%)	41 (97.6%)
- Not remembered	17 (16.3%)	7 (21.6%)	3 (2.8%)	1 (2.4%)
- Refused	11 (10.6%)	1 (2.9%)	-	-
- Missing^2^	-	-	10 (7.0%)	-
Retail Audit method (RA)				
Sales volumes data calculated (excluding negatives)	82 (78.8%)	29 (85.3%)	115 (80.4%)	35 (83.3%)
Sales volumes data calculated (including negatives)^3^	94 (90.4%)	31 (91.2%)	121 (84.6%)	39 (92.9%)
Stock data collected	96 (92.3%)	31 (91.2%)	121 (84.6%)	39 (92.9%)
- Refused	8 (7.7%)	3 (8.8%)	12 (8.4%)	3 (7.1%)
- Missing^2^	-	-	10 (7.0%)	-
Received quantities collected	103 (99.0%)	33 (97.1%)	133 (93.0%)	42 (100.0%)
- Refused	1 (1.0%)	1 (2.9%)	-	-
- Missing^2^	-	-	10 (7.0%)	
Disposed quantities collected	101 (97.1%)	33 (97.1%)	133 (93.0%)	42 (100.0%)
- Refused	3 (2.9%)	1 (2.9%)	-	-
- Missing^2^	-	-	10 (7.0%)	

^1^At the second visit of RA during which RC was implemented. ^2^missing strength data impeded calculation of data in terms of adult equivalent treatment doses. ^3^negative sales volume estimates were obtained when calculating (quantities in stock at 1st visit + quantities received in-between the 2 visits – quantities at 2nd visit – quantities disposed in-between the 2 visits), e.g. quantities stocked at second visit were higher than quantities stocked at first visit although shopkeepers did not report any quantities received. These negative estimates were excluded from the analysis.

At retail outlets, 143 anti-malarial products were surveyed. Sales volume data were collected through the RC for 130 anti-malarial products and through the RA for 115 anti-malarial products (Table 1). For RDTs, 42 different products were surveyed and sales volumes were collected for 41 through the RC and for 35 through the RA (Table 1). Retail sales volumes were estimated through both RC and RA for 113 anti-malarials and 33 RDTs.

RC and RA sales volume estimates were obtained for 34 wholesale outlets when considering anti-malarials and 23 wholesale outlets when considering RDTs. Similarly, estimates were obtained for 58 retail outlets when considering anti-malarials and 33 outlets when considering RDTs. At one retail outlet, the total volume sold was surprisingly high and well above other retailers’ total sales volumes (outlier outlet total sales volume estimated at 129 AETDs compared to volumes at all other retail outlets ranging from 0 to 20 AETDs). This outlying observation obscured the interpretation of results so it was excluded from the main analysis, which was run on paired anti-malarial estimates available for 57 retail outlets.

**Figure 1:**
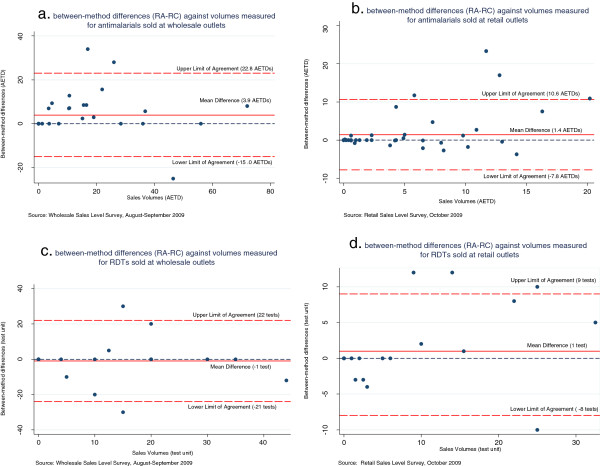
**Scatter plots of the between-method differences (RA-RC) against mean volumes of sales measured. A.** Between-method differences (RA-RC) against volumes measured for anti-malarial sold at wholesale outlets. **B.** Between-method differences (RA-RC) against volumes measured for anti-malarials sold at retail outlets. **C.** Between-method differences (RA-RC) against volumes measured for RDTs sold at wholesale outlets. **D.** Between-method differences (RA-RC) against volumes measured for RDTs sold at retail outlets. RC: recall, RA: retail audit, RDTs: rapid diagnostic tests, AETD: Adult Equivalent Treatment Dose.

Figure 1 presents the scatter plots showing on the y-axis the between-method differences, and on the x-axis the mean of the sales volume obtained by the two methods for the outlet. The dashed blue line drawn at y = 0 represents the line of equality between the volumes measured by the two methods. The mean of the between-method differences is represented by the red line and the LoA between the two methods are represented by the two dashed red lines.

Figure 1a shows no evidence of correlation between the between-method differences and the size of the volumes sold for wholesale anti-malarial sales. This was confirmed by a coefficient of correlation *r* = −0.04 (p = 0.83). The mean difference between RA and RC estimates for anti-malarials was four AETDs indicating that RA provided on average significantly higher estimates than RC (95% CI 0.6-7.2). The LoA indicated that for 95% of paired estimates the between-method difference (RA minus RC) would lie between plus 23 AETDs (95% CI 16.0-28.0) and minus 15 AETDs (95% CI −20.4- -9.0). For RDT sales volumes at wholesale outlets, Figure 1c shows no evidence of correlation between the between-method differences and volumes sold (*r* = 0.04, p = 0.86) and no significant difference between RC and RA estimates (95% CI −6.0-4.0). The LoA were from plus 22 to minus 21 tests.

At retail outlets, there was some indication from Figures 1b and 1d that the between-method differences were positively correlated with volumes sold (for anti-malarials *r* =0.49, p < 0.001; RDTs *r* =0.38, p = 0.03). When including the outlet with the outlying anti-malarial sales volume the evidence of a correlation between the between-method differences and sales volumes was also statistically significant and stronger (*r* = 0.823, p < 0.001; mean difference was two AEDTs (95% CI 0.23-4.0) and LoA were from plus 16 AEDTs to minus 12 AETDs). The mean difference cannot therefore be considered a meaningful estimate of the level of agreement between the two different methods. However, Figures 1b and 1d seem to indicate that RA tended to provide higher estimates than RC at higher levels of sales volumes (around above five AETDs for anti-malarials and 10 units for RDT).

### Qualitative results: fieldworkers’ experiences and perceptions

Data collectors found the RC to be a more convenient approach than RA for collecting sales volume data, notably at retail outlets where shopkeepers said they rarely had customers for malaria treatment. Data collectors also mentioned that retailers seemed to be more comfortable remembering sales volumes of RDTs than anti-malarials and that this was because performing a malaria test was a more memorable and discrete event than selling anti-malarials. However, data collectors often questioned the accuracy of RC sales volume data collected both at retail and wholesale outlets.

*“She said: “it might be like this, [or] it may be like that””.* (Fieldwork diary #1 about RC implemented at a retail outlet).

*“She might have misreported her sales volumes, because I saw five empty boxes of anti-malarials near her”.* (Fieldwork diary #5 about RC implemented at a wholesale outlet).

Data collectors indicated that when shopkeepers could not remember their sales volumes, this was because they often handled other consumer goods, including toiletries or groceries, which were their main selling items. Another reason was that more than one person worked at the shop, making it difficult for respondents to provide accurate estimates. Fieldworkers also said that they perceived wholesalers to be less capable of remembering their sales volumes because they generally handled a wider range of drugs and sold larger volumes.

Data collectors reported that during the RA counting stocks was relatively easy and quick because of the small range of anti-malarials and RDTs available at each outlet. They also reported that counting RDTs tended to be easier than anti-malarials, especially when anti-malarials were kept in opened tins. For example, one interviewer explained that in one shop the tin was not transparent, preventing him from using a ruler so that he had to count each tablet left in the tin. Also, at times, interviewers reported they had estimated more pills in the tin at the second than at the first visit although shopkeepers said that no new tin had been opened. Interviewers said that when they collected data on quantities received and disposed of shopkeepers remembered generally very easily because the reported quantities were generally small and often null. They also reported that shopkeepers were generally surprised to be asked about disposed quantities because they said that they never throw products away nor send these back to suppliers.

However, data collectors reported important challenges around the implementation of RA. First, they indicated that both wholesalers and retailers refused at times to let interviewers physically count the quantities in stock, with this challenge occurring more commonly at wholesale than retail outlets.

*“They did not allow us to count and they did not want to count for us at all […] they said they didn’t want to spend time with us […] they said that it [the survey] was useless and wasting their time”* (Fieldwork diary #40 about RA implemented at a wholesale outlet).

*“She claimed that I asked the same question at first visit. She said that I should write the same amount as at first visit”.* (Fieldwork diary #2 about RA implemented at a wholesale outlet).

Second, data collectors reported that in many cases shopkeepers preferred to estimate their stock from memory, rather than have these counted. Fieldworkers added that this situation was again more common amongst wholesalers who often refused to let interviewers open the cupboards where they kept the drugs.

This was corroborated by the SLS quantitative data, which showed that at SLS1 stocks of anti-malarial drugs were physically counted for around 51% of all anti-malarial products surveyed at wholesale outlets compared to 97% at retail outlets. In outlets where stocks could not be physically counted, quantities stocked were stated by wholesalers. In some cases, data collectors explained that the quantities stocked were estimated by memory due to factors beyond the control of shopkeepers. For example, one wholesaler was said to be refurbishing his shop at first visit so that it was not possible to proceed to the stock count. In other cases, one wholesaler and one retailer did not stock all drugs at the shop premises but at their home so the stock count could not be performed.

As during RC, data collectors questioned the accuracy of the data they had recorded during RA. Data collectors said that in some shops they counted higher quantities at SLS2 than at SLS1, although no new supplies were reportedly received. During a group discussion, a data collector explained that in one wholesale outlet, the shopkeeper had prepared an order at first visit (so quantities were not counted as ‘stocked’) but that a few days later the customer had cancelled the order and the shopkeeper had put the drugs back on the shelves but forgot to consider it as a new quantity received.

Last but not least, fieldworkers reported being worn out by the implementation of RA, because of respondents’ attitudes.

*“She blamed me about what the questions asked”* (Fieldworker #1 during a group discussion).

*“I could hear that she whispered ‘what the hell they come again’ ”* (Fieldworker #1 during a group discussion).

## Discussion

This study compared two methods for measuring commercial outlet sales volumes, the recall and retail audit methods. Before discussing the results, some limitations should be noted. First, the samples were relatively small, notably at the wholesale level where sales volume observations were available for 34 outlets in the case of anti-malarial drugs and for 23 outlets in the case of RDTs. A second limitation is that whilst negative RA estimates clearly indicated data collection errors, and were excluded from the analysis, positive outliers may also have been errors but could not be easily identified. Third, RC estimates that were compared to RA estimates were collected at SLS2, which may have contributed to improving their accuracy as shopkeepers who expected a second interview may have paid more attention to anti-malarial/RDT sales or may have found it easier to identify the recall period because the SLS1 was a memorable event.

At wholesale outlets, the analysis did not allow to conclude that on average the two methods ‘agreed’. The mean difference in anti-malarial sales volume estimates between the two methods was significant and large (four AETDs, 95% CI 0.2-7.2), equivalent to about 30% of mean total sales volumes, and 66% of median total sales volumes. The limits of agreement, which provide an indication of the difference between the measurements at individual outlets, confirmed that estimates obtained through RA and RC methods were often quite different. Overall, for one third of wholesale outlets, the difference between RA and RC estimates represented as much as 50% of the total sales volumes being measured. For RDTs, the analysis of sales volumes showed that the two methods could, on average, be used interchangeably for estimating average sales: the mean difference was small (mean 1 test, 95% CI −6.0 to 4.0), equivalent to about 10% of both mean and median total sales volumes, and overall not statistically significant. However, the study lacked power to estimate the difference with sufficient precision, and the result may be a consequence of the small sample. Overall, RDT sales volume measures varied greatly at individual outlets, with differences between RA and RC of more than 50% of the volumes measured at more than 20% of wholesalers.

At retail outlets, results were more difficult to interpret: bias and limits of agreement were not constant throughout the range of measurements and the between-method differences were positively correlated with volumes, with increasing differences with larger volumes being measured.

Several reasons may explain the between-method differences. At wholesale level, fieldworkers reported that shopkeepers had difficulty remembering anti-malarial sales volumes as they generally stocked a wide range of other products. It is also possible that wholesalers underestimated their sales volumes during recall for fear of disclosure to competitors or regulatory authorities. As indicated in the background section, outlets with a pharmacy license were authorized to wholesale. However, at the time of the study, less than 40% of wholesalers reported holding a pharmacy license allowing them to wholesale medicines [17]. At retail level, the percentage of retailers holding a license allowing them to retail was somewhat higher at 66% [17]. Fieldworkers also experienced some challenges when implementing the RA method, during which it was not always possible to count the quantities stocked. At wholesale outlets, the SLS was implemented at the end of the supply chain survey questionnaire during which wholesalers were also asked about their business characteristics and practices and this may have created fatigue and/or anxiety amongst both respondents and fieldworkers leading to data collection errors. RA estimates might have been affected in some cases by “recall” bias for stock data, and if wholesalers had under-reported their sales volumes through RC they may well have misreported their stocks during RA.

At retail outlets, fieldworkers did not report retailers to have had difficulties remembering their sales volumes but there were concerns about the accuracy of the RC data. Furthermore, the higher estimates produced by RA than RC as sales volumes increased could indicate that retailers may have had more difficulty accurately recalling larger sales volumes than smaller ones. If so, the more accurate measures produced by RA in higher sales volume contexts may justify the additional resources required for implementing this method. Based on project expenditure records, the implementation of RA that required two visits to the same outlet was, as expected, around twice the total cost of RC, amounting to US$8,369, equivalent to US$48 per outlet visit or US$96 per outlet for the two RA visits combined. However, results show that the RA method is also prone to many data collection errors, and is more complex, time-consuming and invasive from the perspective of some commercial providers; these limitations are likely to jeopardize the quality of RA data collected. Stock-outs may have affected the results, with RC estimates being systematically lower than RA as observed at wholesale and retail levels. However, stock-outs may not be an important source of bias in measurement of sales volumes, as generally if a certain drug was not in stock at the time of visit one would expect its sales volumes to be zero. It is possible that sales volumes measured by the RC method may be underestimated if a product was sold during the recall period but was no longer in stock and, therefore, not asked about. Similarly for the RA method, it might be possible to have failed to record sales for a product that was stocked in between the two visits but not during either visit, though this is unlikely.

The finding that the choice of method is likely to dramatically affect the size of the volumes measured has important implications if outlet-specific volumes are needed, for example in the context of an intervention rewarding individual sellers as a function of volume sold. In a study of the market for malaria treatment, this would also have implications if the objective is to measure market size in terms of volumes purchased.

The feasibility and acceptability of different methods is also likely to vary across countries. Providers’ willingness to recall their sales volumes and more generally participate in a medicine outlet survey may be very variable across different sociocultural contexts. For instance, during the ACTwatch supply chain survey, 5% of wholesalers in Cambodia refused to participate whilst in Zambia and Nigeria the refusal rate was 2% and 19% respectively [19,30]. More specifically, whilst anti-malarials represented a small share of wholesale and retail providers’ businesses in Cambodia, larger and highly variable volumes were handled in other ACTwatch countries. For example, the median anti-malarial volumes sold by wholesalers during the week preceding the supply chain survey ranged from 244 AETDs (IQR 26–1,104) in Benin, 689 AETDs (IQR 125–1,933) in Zambia and 1,346 AETDs (IQR 364–4,728) in Nigeria, whilst it was 0 AETD (IQR 0–0.4) in Cambodia. The suitability of different methods for measuring sales volumes is therefore likely to vary across contexts, including the type of outlets (e g, retail *vs* wholesale) and country under study (e g, malaria incidence).

## Conclusion

This study is the first that empirically compares two methods for measuring sales volumes at different anti-malarial outlet types, including both retail and wholesale outlets. It showed that at wholesale outlets the two methods did not agree for most measurements, except for RDT volumes, although the sample size was small for the latter. At retail outlets, the between-method difference was not constant throughout the range of measurement, which made the interpretation of results more difficult. Qualitative research indicated that both RA and RC methods have implementation challenges and demonstrated that the choice of empirical methods in a research project is likely to have important implications for the quality of data to be collected. Whilst the analysis did not provide firm conclusions on which method is more likely to provide more accurate sales estimates, it demonstrated that in Cambodia where sales volumes are relatively small, the RC method appeared to have key advantages: retailers were perceived to easily remember their sales volumes, wholesalers were perceived to find the method less invasive, and fieldworkers found it more convenient. The RC method was also the cheapest to implement. However, as mentioned above, sales volumes in Cambodia are low compared to other malaria-endemic countries and future research should aim to repeat such comparative analysis in contexts where anti-malarial and RDT sales volumes are larger.

## Competing interests

The authors declare that they have no competing interest.

## Authors’ contributions

EP designed the study, led the data collection, conducted the data analysis and wrote the manuscript. IK provided advice during the development of the study design and statistical data analysis. CG provided intellectual guidance on all aspects of the study, including study design, data collection and analysis. IK, BP, ST, CG, KH and KO provided comments and inputs to the manuscript and approved its final version. All co-authors are members of the ACTwatch Study, from which this study draws in terms of methods for sampling retailers and wholesalers, and the design of data collection tools for collecting sales volume data using the recall method. All authors read and approved the final manuscript.
